# Levels and Concentration Ratios of Polychlorinated Biphenyls and Polybrominated Diphenyl Ethers in Serum and Breast Milk in Japanese Mothers

**DOI:** 10.1289/ehp.9032

**Published:** 2006-04-18

**Authors:** Kayoko Inoue, Kouji Harada, Katsunobu Takenaka, Shigeki Uehara, Makoto Kono, Takashi Shimizu, Takumi Takasuga, Kurunthachalam Senthilkumar, Fumiyoshi Yamashita, Akio Koizumi

**Affiliations:** 1 Department of Health and Environmental Sciences, Kyoto University Graduate School of Medicine, Kyoto, Japan; 2 Department of Neurosurgery, Takayama Red Cross Hospital, Takayama, Japan; 3 Department of Obstetrics and Gynecology, Tohoku Kosai Hospital, Sendai, Japan; 4 Kono Obstetrics and Gynecology Clinic, Shizunai, Japan; 5 Shimizu Woman’s Clinic, Takarazuka, Japan; 6 Shimadzu Techno-Research Inc., Kyoto, Japan; 7 Graduate School of Pharmaceutical Sciences, Kyoto University, Kyoto, Japan

**Keywords:** breast milk, partition coefficient, polybrominated diphenyl ethers, polychlorinated biphenyls, quantitative structure—activity relationship, serum

## Abstract

Blood and/or breast milk have been used to assess human exposure to various environmental contaminants. Few studies have been available to compare the concentrations in one matrix with those in another. The goals of this study were to determine the current levels of polybrominated diphenyl ethers (PBDEs) and polychlorinated biphenyls (PCBs) in Japanese women, with analysis of the effects of lifestyle and dietary habits on these levels, and to develop a quantitative structure–activity relationship (QSAR) with which to predict the ratio of serum concentration to breast milk concentration. We measured PBDEs and PCBs in 89 paired samples of serum and breast milk collected in four regions of Japan in 2005. The geometric means of the total concentrations of PBDE (13 congeners) in milk and serum were 1.56 and 2.89 ng/g lipid, respectively, whereas those of total PCBs (15 congeners) were 63.9 and 37.5 ng/g lipid, respectively. The major determinant of total PBDE concentration in serum and milk was the geographic area within Japan, whereas nursing duration was the major determinant of PCB concentration. BDE-209 was the most predominant PBDE congener in serum but not in milk. The excretion of BDE 209 in milk was lower than that of BDE 47 and BDE 153. QSAR analysis revealed that two parameters, calculated octanol/water partition and number of hydrogen-bond acceptors, were significant descriptors. During the first weeks of lactation, the predicted partitioning of PBDE and PCB congeners from serum to milk agreed with the observed values. However, the prediction became weaker after 10 weeks of nursing.

Polybrominated diphenyl ethers (PBDEs) have been found in human breast milk (Darnerud et al. 1998; Noren and Meironyte 1998, 2000). This route is a potential excretion pathway for the mother and a route of exposure to these compounds for the neonate. Thus, the monitoring of breast milk provides data for not only adult exposure but also neonatal exposure.

Recently, an examination of Swedish human milk samples from 1972 to 1997 revealed exponential increases in the concentrations of PBDEs (Darnerud et al. 1998; Noren and Meironyte 1998, 2000). Deca-BDE is used primarily in electrical and electronic applications (e.g., television housing, wire and cable insulation) and to a lesser extent in upholstery textiles. Penta-BDE was formerly used in flexible polyurethane foam for cushions. Octa-BDE was used in acrylonitrile-butadiene-styrene resins intended for business equipment housings. PBDEs are now found as residues in sediment (Song et al. 2004); in marine mammals, fish, and bird eggs (Covaci et al. 2005; Kajiwara et al. 2004; Watanabe et al. 2004); and in the breast milk, serum, whole blood, and adipose tissue of humans (Eslami et al. 2005; Koizumi et al. 2005; Lind et al. 2003; She et al. 2002; Takasuga et al. 2004). In contrast to PBDEs, banning the production and use of polychlorinated biphenyls (PCBs) in the 1970s has decreased PCB serum levels and dietary exposure to PCBs since the 1980s (Koizumi et al. 2005).

The aims of the present study were 2-fold. The first was to determine the current levels of PBDEs and PCBs in Japanese women of reproductive age and to analyze the effects of lifestyle and dietary habits on these levels. The second was to develop a quantitative structure–activity relationship (QSAR) model, which enables us to predict the relationship between serum and breast milk. The second aim addresses the importance of translatability between the serum and milk data.

## Materials and Methods

### Target populations

The present study was approved by the Ethics Committee of the Kyoto University Institutional Review Board, and appropriate written informed consent was obtained from all the participants before sample collection.

After obtaining formal informed consent, we collected blood and breast milk samples from mothers who had delivered and were lactating in maternity hospitals in four regions: Sendai city (population, 1 million) in Miyagi Prefecture, Takarazuka city (population, 250,000) in Hyogo Prefecture, Takayama city (population, 200,000) in Gifu Prefecture, and Shizunai-cho (population, 23,000) in Hokkaido Prefecture.

### Collection of serum samples and breast milk samples

Milk samples were self-collected manually into breast pumps with glass containers at the individual hospitals and transferred to 50-mL polypropylene conical tubes (milk tube) that had been thoroughly rinsed with methanol and acetone before use; samples were kept frozen at –20°C. The target volume was > 20 mL from each mother per sample. Blood samples (10 mL) were collected into two 5-mL vacuum blood collection polypropylene tubes (Venoject II; TERUMO Inc., Tokyo, Japan) (blood tube) from cubital vein by physicians or nurses. The blood and milk samples were shipped within 48 hr to Kyoto University. The serum samples were separated by centrifugation at 3,000*g* for 15 min, transferred to new blood tubes, and stored at –20°C in the Department of Health and Environmental Sciences, Kyoto University Graduate School of Medicine, until analysis.

When the milk samples were collected, we asked the mothers to fill out questionnaires that contained necessary items for milk surveillance (LaKind et al. 2004) and sources of exposure to PBDEs (Ohta et al. 2002; Sakai et al. 2001; Schecter et al. 2005; Wilford et al. 2003), including the duration of lactation, parity, residential history within the previous 5 years, lifestyle and habits, and indoor environment (Supplemental Table 1; available online at http://www.ehponline.org/docs/2006/9032/suppl.pdf).

We prepared eight field blanks per site, each consisting of an empty milk tube and an empty blood tube. In addition, we prepared eight milk tube/blood tube pairs filled with 5 mL of distilled water at the sampling site as field operational blanks. All the blank samples were sent to Kyoto University and run through complete extraction, cleanup, and analysis procedures.

### Serum extraction

The internal standard from mono- to deca-^13^C_12_-PBDE and mono to deca-^13^C_12_-PCB was spiked in the serum (3 g) and extracted by liquid–liquid extraction following the method of Takasuga et al. (2004, 2006). Briefly, in the serum spiked with internal standard, 3 mL ammonium sulfate, 1 mL ethanol, and 2 mL hexane were mixed and extracted twice. The final extract was washed with hexane-washed water, dehydrated with sodium sulfate, and concentrated to 5 mL for further cleanup.

### Milk extraction

The internal standard from mono- through deca-^13^C_12_-PBDE and mono- to deca-^13^C_12_-PCB was spiked in the milk (3 g) and extracted by liquid–liquid extraction. Briefly, in the milk spiked with internal standard, 1 mL saturated potassium oxalate, 2 mL ethanol, 2 mL diethyl ether, and 1 mL hexane were mixed and extracted twice. The final extract was washed with 1 mL of 5% sodium chloride and then dehydrated with sodium sulfate and concentrated to 5 mL for further cleanup.

### Cleanup of serum and milk

The 5-mL extract from serum or milk was subjected to multilayer Florisil silica gel column cleanup (Takasuga et al. 2004, 2006). The multilayer cleaned samples were further concentrated to the injection volume by nitrogen purge.

### Identification and quantification of PBDEs and PCBs

We used high-resolution gas chromatography (HRGC; HP6890, Agilent)/high-resolution mass spectrometry (HRMS; Autospec Ultima; Micromass, Cary, NC, USA) for analysis of PBDEs and PCBs. Details on the HRGC/HRMS program are reported elsewhere (Takasuga et al. 2004, 2006). Briefly, for PBDE analysis we used either a BP-1 [15 m × 0.25 mm i.d. (0.1 μm); SGE Analytical Science Pty. Ltd., Austin, TX, USA] column or a ENV-5MS [15 m × 0.25 mm i.d. (0.1 μm)] column. The column was used with a temperature program of 120°C (1 min), increased 20°C/min to 160°C (0 min), 10°C/min to 260°C (0 min), and 20°C/min to 300°C (8 min). For analysis of PCBs, we used an HT-8 PCB column (60 m × 0.25 mm i.d.; SGE Analytical), which was used with an initial temperature of 150°C (0 min), increased 20°C/min to 200°C (0 min), 5°C/min to 260°C (0 min), and 10°C/min to 300°C (11.5 min). We used an on-column injection program with a 2-μL sample injection volume and with a resolution of *M*/Δ*M* > 10,000 (10% valley). We determined the individual and total concentrations of 13 PBDE congeners [ΣPBDE_13_; International Union of Pure and Applied Chemistry (IUPAC) congeners 15, 28, 47, 99, 100, 153, 154, 183, 196, 197, 206, 207, and 209] and 15 PCB congeners (ΣPCB_15_; IUPAC congeners 74, 99, 118, 138, 146, 153, 156, 163/164, 170, 180, 182/187, 194, 199, 206, and 209).

The limit of detection (LOD) for each PCB congener was 1 pg/g in both serum and breast milk. The LOD of each PBDE congener in serum and milk was between 0.2 and 2 pg/g for di-BDE to hepta-BDE and between 0.3 and 2 pg/g for octa-BDE to deca-BDE. The serum and milk concentrations of PCBs and PBDEs were expressed as nanograms per gram lipid. The lipid content in the serum samples was estimated from the total cholesterol and triglyceride concentrations (Phillips et al. 1989). The lipid content of the milk samples was determined from 2 mL crude extracts by gravimetric method.

### Quality assurance and quality control

PBDE and PCB (native as well as ^13^C_12_−labeled) standard solutions that contained the major congeners of mono-BDE or mono-CB to deca-BDE or deca-CB (> 95% pure) were purchased from Wellington Laboratories (Guelph, Ontario, Canada). The average recovery of individual PBDE congeners was 54–84% in serum (*n* = 100) and 54–103% in milk (*n* = 100), and the average recovery of PCB congeners was 61–79% in serum (*n* = 100) and 68–115% in milk (*n* = 100). The coefficient of variation for each determination was within 15% for both PBDEs and PCBs.

For all field blanks and field operational blanks, all PBDE and PCB congeners were < LOD. Operational blank tubes filled with 5 mL distilled water in an analytical laboratory (Shimadzu Techno-Research Inc., Kyoto, Japan) were also prepared for each eight-sample batch. These operational blanks were < LOD for all PBDE and PCB congeners in both the serum and milk batches. Thus, we did not correct the results for background levels.

### Structure–activity relationship

For the QSAR analysis, we chose congeners that were detected in > 50% of both the serum and milk samples. Theoretical molecular descriptors for the compounds, which included constitutional descriptors, atom-centered fragments, and molecular properties, such as hydrophilicity, molar refractivity, polar surface area, and octanol/water partition coefficient (*K*_ow_), were calculated using Dragon software (version 5.0; Milano Chemo Metrics and QSAR Research Group, Milan, Italy) and ADMET Predictor 1.2.3 (Simulations Plus, Lancaster, CA, USA). The *K*_ow_ calculated by Hansch’s method (CLogP) and the molar refractivity calculated by Hansch’s method (CMR) were calculated using Web applications provided by Daylight Chemical Information Systems (Aliso Viejo, CA, USA). Descriptors that had a bivariate correlation > 0.70 were removed.

We performed a stepwise multiple linear regression analysis using the SAS statistical package (version 8.2; SAS Institute Inc., Cary, NC, USA). All independent variables in the regressions had a significance of at least 95%, based on Student’s *t*-score.

### Statistical analysis

Statistical analyses were conducted after logarithmic transformation of the concentrations of the PBDEs and PCBs. We tested differences between means by analysis of variance (ANOVA) or Student’s *t*-test when appropriate. A stepwise multiple regression analysis was used to explore determinants for the serum and milk levels of contaminants using a forward–backward stepwise regression model (*F*-statistic to enter and stay in the model with a *p*-value of < 0.25). We evaluated the determinants for PBDEs and PCBs in serum and breast milk using a conservative approach based on multiple comparisons of the questionnaire items. Thus, a *p*-value of < 0.01 was considered significant in the multiple regression analysis for the questionnaire items. For the other analyses, a *p*-value of < 0.05 was considered significant. All statistical analyses were carried out with SAS software.

## Results

### Demographic features of the participants

On the whole, there were 20 participants from Hokkaido, 40 from Miyagi, 20 from Gifu, and 9 from Hyogo. The ages of the participants ranged from 20 to 43 years (mean ± SD, 30.1 ± 4.6 years). The results of the questionnaires are summarized in Table 1.

### Determination of PBDEs and PCBs in serum and milk

The concentrations of some congeners in the human samples were < LOD. We treated these samples as 0 pg/g lipid when we calculated the total amount.

**Figure 1:**
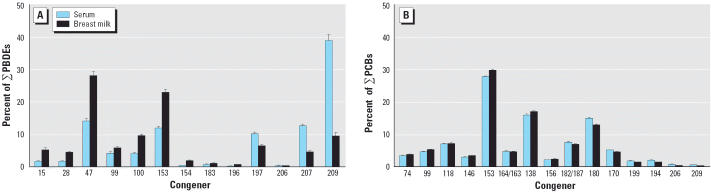
Distributions of PBDE (*A*) and PCB (*B*) congeners in milk and serum from the 89 participants (mean ± SE). The levels of each congener are indicated as the mean percentage of the ΣPBDE or ΣPCB concentration.

The distributions of ΣPBDE_13_ in serum and milk followed log-normal distributions (Kolmogorov-Smirnov-Lilliefors test, *p* > 0.05). The geometric mean (GM) values for the total amounts of ΣPBDE_13_ in the milk and serum samples were 1.56 and 2.89 ng/g lipid, respectively (Table 2). The PBDE congener levels and detection rates for milk and serum are available online (Supplemental Tables 2 and 3, respectively; http://www.ehponline.org/docs/2006/9032/suppl.pdf). BDE-209 was the predominant congener in serum and accounted for 38% of the total PBDEs but was a minor congener in milk and accounted for 8% of the ΣPBDE_13_ (Figure 1A). In milk, BDE-47 and BDE-153 were the major congeners and accounted for 28 and 23% of the total PBDEs, respectively.

The distributions of the ΣPCB_15_ in serum and milk also followed log-normal distributions (Kolmogorov-Smirnov-Lilliefors test, *p* > 0.05). The GM values for ΣPCB_15_ in the milk and serum samples were 63.9 and 37.5 ng/g lipid, respectively (Table 2). The PCB congener levels and detection rates for milk and serum are available online (Supplemental Tables 4 and 5, respectively; http://www.ehponline.org/docs/2006/9032/suppl.pdf). CB-153, CB-138, and CB-180 were the major congeners in both milk and serum (30, 17, and 13% of the total for milk and 28, 16, and 15% of the total for serum, respectively) (Figure 1B).

It should be noted that approximately the same concentrations of the lighter PBDEs (e.g., BDE-47) are present in serum and milk, but BDE-209 is found at 10 times lower concentrations in milk than in serum (Supplemental Tables 2 and 3; available online at http://www.ehponline.org/docs/2006/9032/suppl.pdf). Likewise, almost double the serum concentration of CB-153 is found in milk, whereas more than double the milk concentration of CB-209 is found in serum (Supplemental Tables 4 and 5; available online at http://www.ehponline.org/docs/2006/9032/suppl.pdf).

### Determinants for PCBs and PBDEs in serum and milk

We found significant correlations between ΣPCB_15_ and ΣPBDE_13_ levels in both milk and serum (*r*^2^ = 0.194, *p* < 0.0001 for milk; *r*^2^ = 0.1808, *p* < 0.0001 for serum). There were also significant geographic differences in ΣPBDE_13_ concentrations in milk and serum (ANOVA, *p* = 0.00095 and *p* = 0.00030, respectively; Table 2). The GM for ΣPBDE_13_ in the milk samples was higher for Hokkaido than for the other areas [Tukey’s honest significant difference (HSD) test, *p* < 0.05], whereas the GM for ΣPBDE_13_ in serum samples was higher in Miyagi than in Gifu (Tukey’s HSD test, *p* < 0.05). The PCB levels also exhibited geographic differences (ANOVA, *p* = 0.0029 for milk and *p* < 0.0001 for serum; Table 2). The GMs for ΣPBDE_13_ in both milk and serum samples were higher in Miyagi and Hyogo than in Gifu (Tukey’s HSD test, *p* < 0.05).

Multiple regression analysis revealed that the geographic factor was the primary determinant for the PBDE levels in both milk and serum (data not shown). In contrast, nursing duration was the significant determinant for PCB levels in both serum and milk (data not shown). To investigate the possible association between hospitals and nursing durations, we tested whether nursing duration was a determinant for PBDE or PCB levels within a single hospital. The nursing duration was correlated with the ΣPBDE_13_ in serum in Miyagi (*n* = 38, Kendall’s τ= −0.266, *p* = 0.0187) and the ΣPCB_15_ in both serum and milk in Miyagi (*n* = 38, Kendall’s τ= –0.426, *p* = 0.0002, and Kendall’s τ= –0.312, *p* = 0.0059, respectively; data not shown).

### QSAR analysis

BDE-154, BDE-183, BDE-196, and BDE-206 were eliminated from the analysis because of their low detection rates in serum and/or milk (< 50%). In the first step, we calculated the mean ratios of milk concentrations (nanograms per gram lipid) to serum concentrations (nanograms per gram lipid) for individual congeners from milk and serum as surrogates for their partition coefficients (Supplemental Table 6; available online at http://www.ehponline.org/docs/2006/9032/suppl.pdf). Using these mean ratios, we then applied a multiple linear regression analysis using various descriptors for individual PCB and PBDE congeners. The descriptors that have been used for QSAR analysis include hydrophobicity [log *K*_ow,_ CLogP, (octanol/water partition coefficient calculated by Hansch’s method), and MLogP (octanol/water partition coefficient calculated by Moriguchi’s method)], size [MW (molecular weight) and MgVol (molar volume calculated by McGowan’s method)], polarizability [CMR, (molar refractivity calculated by Hansch’s method), AMR (calculated by Ghose and Crippen’s method), and PolarizG (polarizability calculated by Glen’s method)], and constitutional descriptors [TPSA (topologic polar surface area), HBA (number of hydrogen-bond acceptors), nCL (number of chlorines), and nBR (number of bromines)] (Table 3) (Abraham and McGowan 1987; Ghose and Crippen 1987; Glen 1994; Leo et al. 1971; Moriguchi et al. 1994).

Table 3 summarizes the correlation coefficients between pairs of the descriptors, together with regression coefficients for each descriptor. Regarding PCB and PBDE congeners, the descriptors for hydrophobicity (log *K*_ow_, CLogP, and MLogP), molecular size (MW and MgVol), and polarizability (CMR, AMR, and PolarizG) were collinear, and each correlated well with the milk/serum partition coefficient (log *P*). We explored the combination of the descriptors that exhibited the highest multiple regression coefficient (*r*) and obtained the following equation:


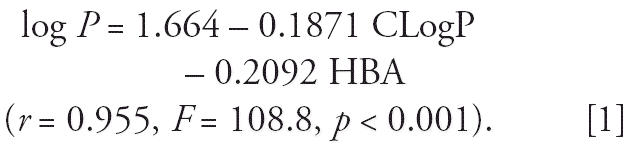


**Figure 2:**
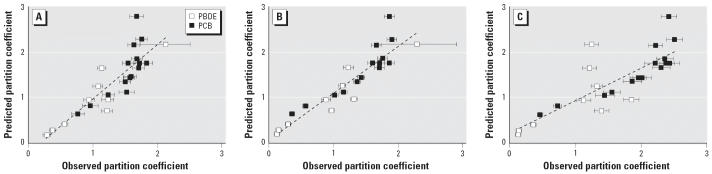
Predicted and observed partition coefficients (milk/serum) of PBDE and PCB congeners by nursing duration. (*A*) Weeks 0–1. (*B*) Weeks 2–10. (*C*) Weeks 11–88. The *y*-axis represents the predicted partition coefficient, and the *x*-axis represents the ratio of the observed milk concentration to the observed serum concentration (mean ± SE). For (*A*), the relationship between the predicted (*y*) and observed (*x*) partition coefficients was *y* = 1.210*x* – 0.237 (*r* = 0.866, *p* < 0.001, *n* = 26); for weeks 2–10 (*B*), *y* = 1.028*x* + 0.082 (*r* = 0.921, *p* < 0.001, *n* = 38); for weeks 11–88 (*C*), *y* = 0.717*x* + 0.233 (*r* = 0.824, *p* < 0.001, *n* = 25).

Because partition coefficients have been reported to be dependent on the nursing period (LaKind et al. 2004), we tested the relationship between the predicted and observed mean partition coefficients for three nursing durations (Figure 2). For nursing durations ≤10 weeks, the partition coefficients predicted by the QSAR analysis agreed with the observed values. However, the coefficient of *x* was smaller for nursing durations > 10 weeks, suggesting that the prediction became weaker for longer nursing periods.

## Discussion

In this article we have reported the current levels of ΣPBDE_13_, including deca-BDE (BDE-209), in serum and milk from Japanese mothers. We found that BDE-209 was the most abundant congener in serum but a minor congener in milk. Its abundance in serum suggests that wide industrial use of BDE-209 may result in exposure (Watanabe and Sakai 2003). Thus, low partitioning of this congener from serum to milk might have resulted in the underestimation of human adult exposure to deca-BDE, if the exposure monitoring system used was dependent solely on milk surveillance.

Table 4 shows the recent data on PBDEs in breast milk and serum from 12 countries. The current total PBDE levels in Japan are significantly lower than those in most Western countries (Kalantzi et al. 2004; López et al. 2004; Mazdai et al. 2003; Morland et al. 2005; Pereg et al. 2003; Ryan and Patry 2000; Ryan and van Oostdam 2004; Schecter et al. 2003; She et al. 2004; Sjödin et al. 2004) and appear to be approximately equal to those of Sweden (Guvenius et al. 2003; Kalantzi et al. 2004; Lind et al. 2003; Sjödin et al. 1999), Spain (Schuhmacher et al. 2004), Italy (Ingelido et al. 2004), Germany (Vieth et al. 2004), and Finland (Strandman et al. 2000). Even for BDE-209, exposure was relatively lower in Japan than in the United States and Mexico. Even taking into account the variations in the measured PBDE congeners, the above argument holds true.

We investigated factors that may influence the PBDE or PCB levels in serum and milk. We found that the geographic factor was the major determinant of PBDE levels in Japan. In contrast, current nursing duration was most significant for PCBs. Because the current nursing duration was confounded by the variation in the timing of milk collection in the different hospitals, one could argue that the apparent differences might be explained partly by the geographic factor. However, the current nursing duration remained significant for both PBDEs and PCBs even within sample series from a single hospital, indicating that their concentrations became lower as the nursing period became longer, as previously reported by others (Wilson et al. 1985).

Human milk or serum surveillance is typically performed to monitor temporal changes in the concentrations of environmental chemicals or to compare the concentrations of environmental chemicals among different populations. However, only a few trials to bridge the values for serum and milk have been carried out for environmental chemicals (Greizerstein et al. 1999). In contrast, there have been several models and methods for predicting drug transfer into human milk (Fleishaker 2003) using the QSAR approach. We applied the same approach for PCBs and PBDEs. The analysis revealed that CLogP and HBA are sufficient predictors of the transfer from serum to milk. For PBDEs, the oxygen atom bridging two halogenated aryl groups, which functions as a hydrogen-bond acceptor, appeared to reduce the transfer from serum to milk. On the other hand, the model only weakly predicted the partition coefficients in the later stages of nursing (≥11 weeks), as suggested by Wilson et al. (1985). With the limitation of the nursing period as a mode of prediction by Equation 1, the present model can be practically used for translating the concentrations in the two samples.

## Conclusion

BDE-209 was the PBDE detected at the highest concentration in serum of Japanese lactating women, but its excretion in milk was lower than that of the lower brominated diphenyl ethers BDE-47 and BDE-153. Geographic location within Japan and the duration of nursing were discernible determinants for levels of PBDEs and PCBs in human serum and milk, respectively. The levels of PBDEs in Japan were much lower than those in the United States, Canada, and Mexico but similar to those in European countries. The application of QSAR for the structure–partition relationship revealed that the values for serum and milk are translatable to each other.

## Figures and Tables

**Table 1:** Characteristics of the participants.

	Total	Hokkaido	Miyagi	Gifu	Hyogo	*p*-Value
No. of participants	89	20	40	20	9	
Age (years)						
20–29	45	13	19	10	3	0.66
30–39	40	6	20	9	5	
40–49	4	1	1	1	1	
Mean ± SD	30.1 ± 4.6	27.7 ± 4.8	30.7 ± 4.1	30.0 ± 4.3	33.3 ± 4.5	0.01
Parity (mean ± SD)	1.45 ± 0.6	1.55 ± 0.8	1.33 ± 0.5	1.55 ± 0.7	1.56 ± 0.5	0.43
Nursing week at milk collection (mean ± SD)	13.6 ± 22.1	1.55 ± 1.6	12.0 ± 18.6	33.4 ± 30.3	3.11 ± 0.9	< 0.0001*
Occupation [no. (%)]						
Housewife	50 (56.2)	13 (65.0)	21 (52.5)	9 (45.0)	7 (77.8)	0.21
Office worker	16 (18.0)	1 (5.0)	11 (27.5)	4 (20.0)	0	
Technical professional	22 (24.7)	5 (25.0)	8 (20.0)	7 (35.0)	2 (22.2)	
Farmer	1 (1.1)	1 (5.0)	0	0	0	
Use electronic equipment [no. (%)]						
Personal computer
Frequent use	43 (48.3)	4 (20.0)	27 (67.5)	8 (40.0)	4 (44.4)	0.004*
Rare use	46 (51.7)	16 (80.0)	13 (32.5)	12 (60.0)	5 (55.6)	
Mobile phone						
Frequent use	58 (65.2)	13 (65.0)	26 (65.0)	15 (75.0)	4 (44.4)	0.47
Rare use	31 (34.8)	7 (35.0)	14 (35.0)	5 (25.0)	5 (55.6)	
Television						
Frequent use	69 (77.5)	17 (85.0)	29 (72.5)	17 (85.0)	6 (66.7)	0.36
Rare use	20 (22.5)	3 (15.0)	11 (27.5)	3 (15.0)	3 (33.3)	
Household furnishings [no. (%)]						
Carpet						
Frequent use	65 (73.0)	18 (90.0)	28 (70.0)	12 (60.0)	7 (77.8)	0.14
Rare use	24 (27.0)	2 (10.0)	12 (30.0)	8 (40.0)	2 (22.2)	
Cushions						
Frequent use	52 (58.4)	10 (50.0)	24 (60.0)	10 (50.0)	8 (88.9)	0.15
Rare use	37 (41.6)	10 (50.0)	16 (40.0)	10 (50.0)	1 (11.1)	
Sofa						
Frequent use	66 (74.2)	18 (90.0)	30 (75.0)	11 (55.0)	7 (77.8)	0.08
Rare use	23 (25.8)	2 (10.0)	10 (25.0)	9 (45.0)	2 (22.2)	
Curtains						
Frequent use	81 (91.0)	18 (90.0)	37 (92.5)	17 (85.0)	9 (100.0)	0.46
Rare use	8 (9.0)	2 (10.0)	3 (7.5)	3 (15.0)	0 (0.0)	
Blinds						
Frequent use	42 (47.2)	10 (50.0)	19 (47.5)	9 (45.0)	4 (44.4)	0.99
Rare use	47 (52.8)	10 (50.0)	21 (52.5)	11 (55.0)	5 (55.6)	
Fish consumption (> once/week) [no. (%)]						
Yellowtail and young yellowtail						
Yes	14 (15.7)	0 (0.0)	4 (10.0)	8 (40.0)	2 (22.2)	0.003*
No	75 (84.3)	20 (100.0)	36 (90.0)	12 (60.0)	7 (77.8)	
Mackerel						
Yes	34 (38.2)	5 (25.0)	12 (30.0)	10 (50.0)	7 (77.8)	0.03
No	55 (61.8)	15 (75.0)	28 (70.0)	10 (50.0)	2 (22.2)	
Salmon						
Yes	56 (62.9)	13 (65.0)	30 (75.0)	9 (45.0)	4 (44.4)	0.30
No	33 (37.1)	7 (35.0)	10 (25.0)	11 (55.0)	5 (58.6)	
Smoking status [no. (%)]						
Nonsmoker	56 (62.9)	10 (50.0)	27 (67.5)	11 (55.0)	8 (88.9)	0.17
Ex-smoker	25 (28.1)	8 (40.0)	10 (25.0)	7 (35.0)	0 (0.0)	
Current smoker	4 (4.5)	2 (10.0)	1 (2.5)	1 (5.0)	0 (0.0)	
Passive smoker	4 (4.5)	0 (0.0)	2 (5.0)	1 (5.0)	1 (11.1)	
Alcohol consumption [no. (%)]						
Nondrinker	35 (39.3)	12 (60.0)	12 (30.0)	7 (35.0)	4 (44.4)	0.21
Ex-drinker	48 (53.9)	7 (35.0)	26 (65.0)	10 (50.0)	5 (55.6)	
Current drinker	6 (6.7)	1 (5.0)	2 (5.0)	3 (15.0)	0 (0.0)	

* *p* < 0.01; *p-*values were calculated for continuous values by ANOVA and for categorical values for the chi-square test or Fisher’s exact test.

**Table 2:** Concentrations (ng/g lipid) of PBDEs or PCBs in human milk or serum samples.

Measure/area	No. of participants	GM (GSD)*a*	Mean ± SD	Range	Q25	Median	Q75
PBDE in milk							
Hokkaido	20	2.23 (1.47)^A^	2.39 ± 0.94	1.02–4.55	1.72	2.22	2.97
Miyagi	40	1.42 (1.56)^B^	1.55 ± 0.65	0.49–3.11	1.06	1.46	1.98
Gifu	20	1.45 (1.51)^B^	1.58 ± 0.71	0.82–3.30	1.01	1.40	2.00
Hyogo	9	1.30 (1.65)^B^	1.45 ± 0.70	0.66–2.38	0.83	1.31	2.31
Total	89	1.56 (1.59)	1.74 ± 0.81	0.49–4.55	1.13	1.54	2.24
PBDE in serum							
Hokkaido	20	2.75 (1.47)^AB^	2.93 ± 1.04	1.04–5.43	2.24	2.96	3.50
Miyagi	40	3.64 (1.66)^B^	4.21 ± 3.14	1.33–21.19	2.68	3.56	4.93
Gifu	20	2.06 (1.55)^A^	2.24 ± 0.92	0.74–4.50	1.45	2.34	2.71
Hyogo	9	2.52 (1.76)^AB^	2.84 ± 1.32	0.76–5.38	1.78	3.13	3.41
Total	89	2.89 (1.68)	3.34 ± 2.37	0.74–21.19	2.16	2.99	3.76
PCB in milk							
Hokkaido	20	58.91 (1.53)^AB^	64.50 ± 29.91	20–160	50.0	60.0	71.0
Miyagi	40	70.75 (1.56)^B^	78.48 ± 40.66	29–250	54.5	72.5	89.3
Gifu	20	47.24 (1.76)^A^	54.95 ± 30.17	18–130	33.3	51.5	72.0
Hyogo	9	94.64 (1.75)^B^	109.44 ± 58.41	39–190	65.0	93.0	170.0
Total	89	63.86 (1.69)	73.18 ± 40.90	18–250	47.0	65.0	88.0
PCB in serum							
Hokkaido	20	35.92 (1.61)^AB^	40.65 ± 24.49	14–130	29.8	35.0	49.0
Miyagi	40	45.80 (1.72)^B^	53.00 ± 31.24	15–170	32.8	51.0	62.3
Gifu	20	22.26 (1.88)^A^	27.25 ± 18.86	7.9–82	14.0	22.0	35.5
Hyogo	9	54.32 (1.85)^B^	65.22 ± 40.67	23–130	34.0	50.0	89.0
Total	89	37.52 (1.89)	45.67 ± 30.58	7.9–170	26.0	38.0	57.0

Abbreviations: GSD, geometric SD; Q25, first quartile; Q75, third quartile.

a Different letters (A, B, or AB) indicate that the corresponding values are statistically different by Tukey’s HSD test after ANOVA (p < 0.05).

**Table 3:** Correlation coefficients between pairs of molecular descriptors or log *P* for PCBs and PBDEs.

	log *K*_ow_	CLogP	MLogP	MW	MgVol	CMR	AMR	PolarizG	TPSA	HBA	nCL	nBR
log *K*_ow_	1											
CLogP	0.978	1										
MLogP	0.899	0.948	1									
MW	0.876	0.835	0.634	1								
MgVol	0.900	0.866	0.677	0.998	1							
CMR	0.967	0.958	0.832	0.956	0.972	1						
AMR	0.968	0.960	0.836	0.954	0.970	1.000	1					
PolarizG	0.964	0.954	0.823	0.961	0.975	1.000	1.000	1				
TPSA	0.270	0.189	–0.125	0.667	0.631	0.437	0.430	0.450	1			
HBA	0.270	0.189	–0.125	0.667	0.631	0.437	0.430	0.450	1.000	1		
nCL	–0.102	0.008	0.305	–0.540	–0.490	–0.270	–0.263	–0.286	–0.936	–0.936	1	
nBR	0.648	0.570	0.301	0.928	0.903	0.778	0.773	0.788	0.871	0.871	–0.816	1
log *P*	–0.891	–0.894	–0.731	–0.921	–0.933	–0.940	–0.939	–0.941	–0.499	–0.499	0.326	–0.777

**Table 4:** PBDE levels in human milk and blood samples from different countries.

			ΣPBDE (ng/g lipid)				
Country/type	No. of samples	Year of sampling	Mean	Median	BDE-209mean	PBDE congeners included in ΣPBDE	Reference
Japan							
Milk	105	2004	2.54	1.28		28, 47, 99, 100, 153, 154	Eslami et al. 2005
Milk	89	2005	1.74	1.54	0.12	15, 28, 47, 99, 100, 153, 154, 183, 196, 197, 206, 207, 209	Present study
Serum	40	1995	1.8	1.3		47, 99, 100, 153	Koizumi et al. 2005
Serum	89	2005	3.34	2.99	1.20	15, 28, 47, 99, 100, 153, 154, 183, 196, 197, 206, 207, 209	Present study
Milk	12	1999	1.72			28, 47, 99, 153, 154	Ohta et al. 2002
Milk	1(27)^a^	2000	1.39		0.04	28, 37, 47, 66, 75, 77, 85, 99, 100, 138, 153, 154, 183	Akutsu et al. 2003
Blood	156	1999–2001	13	6.9	9.20	3, 7, 15, 17, 28, 47, 49, 66, 71, 77, 85, 99, 100, 119, 126, 138, 139, 153, 154, 183, 209	Takasuga et al. 2004
Milk	4	2003	1.04			17, 25, 28, 30, 32, 33, 35, 37, 47, 49, 66, 71, 75, 77, 85, 99, 100, 116, 119, 126, 138, 153, 154, 155, 166	Hirai et al. 2004
Blood	4	2003	0.3			17, 25, 28, 30, 32, 33, 35, 37, 47, 49, 66, 71, 75, 77, 85, 99, 100, 116, 119, 126, 138, 153, 154, 155, 166	Hirai et al. 2004
United States							
Milk	47	2002	73.9	34	0.92	28, 47, 99, 100, 153, 154	Schecter et al. 2003
Milk	16	2004	77.5	48.5	0.38	28, 32, 33, 47, 66, 71, 85, 99, 100, 153, 154, 183, 209	She et al. 2004
Serum	93	2001–2003	24.6			47, 85, 99, 100, 153, 154, 183	Morland et al. 2005
Serum	7	2000–2002		61		17, 28, 47, 66, 85, 99, 100, 153, 154, 183, 203, 209	Sjödin et al. 2004
Serum	12	2001		37		47, 99, 100, 153, 154, 183	Mazdai et al. 2003
Canada							
Milk	10	1992	5.65	3.03		28, 47, 99, 100, 153	Ryan and Patry 2000
Milk	98	2001–2002	22			28, 47, 99, 100, 153	Pereg et al. 2003
Plasma	10	1994–1999	23.3	20.3		28, 47, 85, 99, 100, 153, 154, 183	Ryan and van Oostdam 2004
Mexico							
Milk	7	2003	4.4		0.30	47, 99, 100, 153, 154, 209	López et al. 2004
Plasma	5	2003	29.1		9.50	47, 99, 100, 153, 154, 209	López et al. 2004
United Kingdom							
Milk	54	2001–2003	8.9	6.3		17, 28, 32, 35, 37, 47, 49, 71, 75, 85, 99, 100, 119, 153, 154	Kalantzi et al. 2004
Sweden							
Milk	93	1996–1999	4.01	3.15		47, 99, 100, 153, 154	Lind et al. 2003
Serum	20	1997		3.3		47, 153, 154, 183, 209	Sjödin et al. 1999
Milk	15	2000–2001		2.14		17, 28, 47, 66, 85, 99, 100, 153, 154, 183	Guvenius et al. 2003
Plasma	15	2000–2001		2.07		17, 28, 47, 66, 85, 99, 100, 153, 154, 183	Guvenius et al. 2003
Norway							
Serum	1(29)^a^	1999	3.34			28, 47, 99, 100, 153, 154	Thomsen et al. 2002
Finland							
Milk	11	1994–1998	2.25	1.62		28, 47, 99, 153	Strandman et al. 2000
Germany							
Milk	93	2001–2003	2.23	1.78	0.17	28, 47, 99, 153, 154, 183, 209	Vieth et al. 2004
Netherlands							
Serum	78	2001–2002	10.7	9.3		47, 99, 100, 153, 154	Weiss et al. 2004
Spain							
Milk	15	2002	2.41	1.7		15 congeners	Schuhmacher et al. 2004
Italy							
Milk	4(40)*^a^*	2000–2001	2.75			28, 47, 66, 85, 99, 100, 138, 153, 154, 183	Ingelido et al. 2004

a The numbers of pooled samples are shown in parentheses.
