# Supplementary material for: Levels and Concentration Ratios of Polychlorinated Biphenyls and Polybrominated Diphenyl Ethers in Serum and Breast Milk in Japanese Mothers
Source: Environ Health Perspect. 2006 Apr 18;114(8):1179–85. doi: 10.1289/ehp.9032 (PMC1552037; doi:10.1289/ehp.9032)
Supplement: Supplemental Figures and Tables [file ehp0114-001179s1.pdf]

Supplementary table 1. Questionnaire

An exposure assessment questionnaire for polybrominated diphenyl ethers: the nursing mother study

Age	( )
Present city of residence	( )
Numbers of delivery	( )
Nursing period (week)	( )

Please answer the following questions. We appreciate your cooperation in taking the time to fill in the answers. We will later statistically analyze them in order to search for any possible predictors for the levels of polybrominated diphenyl ethers present in your serum or breast milk.

1. Please describe briefly your residential history within the last 5 years as is shown in the example. If you have lived in another country or countries, please describe the names of the particular cities, with the duration and time period of residence.

City	Time period	Duration of residence
e.g. Hyogo Nishinomiya	2000-2002	2y6m
New York, USA	2002-2004	2y
Shanghai, China	2004-2004	4m
Kyoto	2005-present	2m
______	______	______
______	______	______
______	______	______
______	______	______

2. Please describe your occupational history.

e.g. Household wife	2000-present
Office worker	1996-2000
______	______
______	______
______	______

3. Please describe how many hours per day you ordinarily use the following household electrical appliances.

A. Personal computer:

1. I use it for (   ) hours per day in the last (   ) years.
2. I seldom use it.

B. Mobile phone

1. I use it for (   ) hours per day in the last (   ) years.
2. I seldom use it.

C. Television

1. I watch it for (   ) hours per day in the last (   ) years.
2. We do not have TV.

D. Other household electric appliance

D1. ______________ (   ) hours per day in recent (   ) years.
D2. ______________ (   ) hours per day in recent (   ) years.
D3. ______________ (   ) hours per day in recent (   ) years.
D4. ______________ (   ) hours per day in recent (   ) years.
D5. ______________ (   ) hours per day in recent (   ) years.

E. Have you been ordinarily using the following furniture or items in the home or at work?

Carpet

At home for (   ) years.
At work for (   ) years.

Cushions

At home for (   ) years.
At work for (   ) years.

Sofa

At home for (   ) years.
At work for (   ) years.

Curtains

At home for (   ) years.

At work for (   ) years.

Blinds

At home for (   ) years.

At work for (   ) years.

F. Please check which fish you eat regularly.

Yellow tail	( )	times per week
Horse Mackerel or Mackerel	( )	times per week
Salmon	( )	times per week

Other fish,

1. ______________________	( )	times per week
2. ______________________	( )	times per week
3. ______________________	( )	times per week

G. Please describe any medicine(s) or supplement(s) you routinely take.

	Medicine or supplement	Frequency
e.g.	Aspirin	Once a month
	Anti-histamine	Every spring season for pollen allergy
	Vitamin compound	Every day
	______________________	____
	______________________	____
	______________________	____

H. Please check your smoking and drinking status

Smoking:

( ) I have never smoked.

( ) I have never smoked but am passively exposed.

( ) I am an ex-smoker

and had smoked ( ) cigarettes/cigars per day

for ( ) years until the age of ( ) years.

( ) I am a current smoker.

I smoke ( ) cigarettes/cigars per day

for ( ) years until the age of ( ) years.

Drinking:

( ) I have never been a drinker.

( ) I am an ex-drinker

and had drunk ( ) ml / week of [check one : beer, sake, whiskey, wine or other ( )] for ( ) years from the age of ( ) to the age of ( ) years.

( ) I am a current drinker

and drink ( ) ml / week of [check one : beer, sake, whisky, wine or other ( )] for ( ) years.

Thank you for your cooperation. If you have any comments, please describe them below.

____

Supplementary table 2. Concentrations of each congener of PBDE in human milk samples (ng/g lipid)

Area	No. of participants	DiBDE #15 GM (GSD)	TrBDE #28 GM (GSD)	TeBDE #47 GM (GSD)	PeBDE #99 GM (GSD)	PeBDE #100 GM (GSD)	HxBDE #153 GM (GSD)	HxBDE #154 GM (GSD)	HpBDE #183 GM (GSD)	OBDE #196 GM (GSD)	OBDE #197 GM (GSD)	NBDE #206 GM (GSD)	NBDE #207 GM (GSD)	DeBDE #209 GM (GSD)
Hokkaido	20	0.13 (3.00)	0.07 (1.56)	0.35 (1.55)	0.10 (1.46)	0.14 (1.58)	0.39 (1.60)	0.03 (1.60)	0.03 (1.64)	0.03 (1.67)	0.21 (1.65)	0.05 (1.53)	0.19 (1.68)	0.40 (1.76)
Miyagi	40	0.05 (2.00)	0.06 (1.77)	0.43 (1.86)	0.08 (1.75)	0.14 (1.67)	0.33 (1.52)	0.02 (1.76)	0.01 (1.57)	0.01 (1.61)	0.07 (1.65)	0.04 (1.58)	0.04 (1.85)	0.08 (2.26)
Gifu	20	0.04 (2.45)	0.06 (1.69)	0.45 (1.71)	0.09 (1.91)	0.15 (1.52)	0.29 (1.52)	0.03 (1.49)	0.01 (1.64)	0.01 (1.58)	0.08 (1.36)	0.02 (1.00)	0.05 (1.46)	0.10 (1.57)
Hyogo	9	0.05 (1.98)	0.07 (1.90)	0.37 (1.74)	0.07 (1.28)	0.13 (1.98)	0.36 (1.81)	0.02 (1.79)	0.01 (1.60)	0.01 (1.32)	0.05 (1.48)	N.D.	0.03 (1.24)	0.06 (1.94)
Total	89	0.06 (2.61)	0.07 (1.74)	0.41 (1.77)	0.08 (1.72)	0.14 (1.66)	0.34 (1.56)	0.03 (1.68)	0.02 (1.75)	0.01 (1.93)	0.09 (1.95)	0.04 (1.72)	0.06 (2.34)	0.12 (2.61)
(% of detected samples)		98.9	100.0	100.0	100.0	100.0	100.0	100.0	97.8	67.4	100.0	11.2	95.5	92.1

N.D.: Under the LOD for all samples.

Supplementary table 3. Concentrations of each congener of PBDE in human serum samples (ng/g lipid)

Area	No. of participants	DiBDE #15 GM (GSD)	TrBDE #28 GM (GSD)	TeBDE #47 GM (GSD)	PeBDE #99 GM (GSD)	PeBDE #100 GM (GSD)	HxBDE #153 GM (GSD)	HxBDE #154 GM (GSD)	HpBDE #183 GM (GSD)	OBDE #196 GM (GSD)	OBDE #197 GM (GSD)	NBDE #206 GM (GSD)	NBDE #207 GM (GSD)	DeBDE #209 GM (GSD)
Hokkaido	20	0.06 (2.15)	0.06 (1.31)	0.33 (1.32)	0.10 (1.38)	0.11 (1.58)	0.31 (1.46)	0.05 (1.15)	0.06 (1.20)	0.11(1.00)	0.32 (1.54)	N.D.	0.42 (1.42)	1.12 (1.35)
Miyagi	40	0.06 (1.48)	0.07 (1.39)	0.43 (1.61)	0.12 (1.92)	0.13 (1.73)	0.35 (1.64)	0.07 (1.58)	0.06 (1.23)	0.11 (1.40)	0.26 (1.45)	0.40 (1.15)	0.35 (1.58)	1.75 (2.01)
Gifu	20	0.05 (2.75)	0.06 (1.81)	0.31 (1.41)	0.08 (1.23)	0.09 (1.39)	0.23 (1.38)	0.05 (1.12)	0.06 (1.30)	0.09 (0.00)	0.25 (1.59)	N.D.	0.32 (1.35)	0.64 (1.87)
Hyogo	9	0.05 (1.76)	0.07 (1.45)	0.33 (1.44)	0.08 (1.25)	0.11 (1.75)	0.35 (1.83)	0.05 (1.00)	0.06 (1.08)	N.D.	0.22 (1.35)	0.32 (1.00)	0.27 (1.13)	1.10 (1.84)
Total	89	0.05 (1.74)	0.07 (1.39)	0.37 (1.56)	0.10 (1.70)	0.11 (1.71)	0.31 (1.67)	0.06 (1.50)	0.06 (1.50)	0.11 (1.34)	0.27 (1.51)	0.38 (1.17)	0.35 (1.52)	1.20 (2.05)
(% of detected samples)		69.7	68.5	100.0	98.9	93.3	100.0	18.0	40.4	6.7	98.9	4.5	98.9	92.1

N.D.: Under the LOD for all samples.

Supplementary table 4. Concentrations of each congener of PCB in human milk samples (ng/g lipid)

Area	No. of participants	TeCB #74 GM (GSD)	PeCB #99 GM (GSD)	PeCB #118 GM (GSD)	HxCB #146 GM (GSD)	HxCB #153 GM (GSD)	HxCB#164/#163 GM (GSD)	HxCB#138 GM (GSD)	HxCB#156 GM (GSD)	HpCB#182/#187 GM (GSD)	HpCB #180 GM (GSD)	HpCB #170 GM (GSD)	OCB #199 GM (GSD)	OCB#194 GM (GSD)	NCB #206 GM (GSD)	DeCB #209 GM (GSD)
Hokkaido	20	2.30 (1.56)	3.02 (1.04)	4.15 (1.59)	2.00 (1.54)	16.90 (1.55)	2.40 (1.56)	10.02 (1.50)	1.31 (1.64)	4.02 (1.64)	7.43 (1.67)	2.73 (1.60)	0.85 (1.69)	0.80 (1.80)	0.25 (1.64)	0.21 (1.63)
Miyagi	40	2.36 (1.75)	3.67 (1.66)	4.87 (1.72)	2.28 (1.59)	21.40 (1.58)	3.24 (1.65)	12.09 (1.63)	1.63 (1.63)	4.58 (1.56)	8.68 (1.55)	3.14 (1.56)	0.88 (1.54)	0.85 (1.54)	0.19 (1.56)	0.09 (1.67)
Gifu	20	1.60 (2.07)	2.34 (1.74)	3.19 (1.79)	1.55 (1.84)	13.84 (1.81)	2.11 (1.88)	7.97 (1.79)	1.01 (1.95)	3.30 (1.70)	6.35 (1.70)	2.18 (1.70)	0.63 (1.58)	0.69 (1.61)	0.15 (1.51)	0.07 (1.48)
Hyogo	9	4.02 (1.92)	4.41 (2.12)	6.52 (2.07)	3.69 (1.75)	27.35 (1.79)	4.88 (1.60)	13.69 (1.96)	2.26 (1.55)	6.83 (1.69)	11.76 (1.60)	4.27 (1.58)	1.18 (1.58)	1.15 (1.60)	0.20 (1.47)	0.08 (1.39)
Total	89	2.27 (1.89)	3.23 (1.75)	4.40 (1.81)	2.13 (1.75)	18.86 (1.72)	2.87 (1.78)	10.69 (1.73)	1.44 (1.79)	4.30 (1.69)	8.06 (1.67)	2.89 (1.67)	0.84 (1.64)	0.83 (1.65)	0.19 (1.61)	0.10 (1.84)
(% of detected samples)		100	100	100	100	100	100	100	100	100	100	100	100	100	98.9	96.6

Supplementary table5. Concentrations of each congener of PCB in human serum samples (ng/g lipid)

Area	No. of	TeCB #74	PeCB #99	PeCB #118	HxCB #146	HxCB #153	HxCB #164/#163	HxCB #138	HxCB #156	HpCB #182/#187	HpCB #180	HpCB #170	OCB #199	OCB #194	NCB #206	DeCB #209
	participants	GM (GSD)	GM (GSD)	GM (GSD)	GM (GSD)	GM (GSD)	GM (GSD)	GM (GSD)	GM (GSD)	GM (GSD)	GM (GSD)	GM (GSD)	GM (GSD)	GM (GSD)	GM (GSD)	GM (GSD)
Hokkaido	20	1.40 (1.59)	1.87 (1.57)	2.57 (1.65)	1.06 (1.62)	10.03 (1.65)	1.59 (1.70)	6.12 (1.55)	0.76 (1.73)	2.44 (1.74)	4.61 (1.74)	1.68 (1.69)	0.50 (1.78)	0.62 (1.71)	0.27 (1.54)	0.24 (1.40)
Miyagi	40	1.38 (1.95)	1.92 (1.83)	2.99 (1.90)	1.33 (1.79)	12.62 (1.75)	2.09 (1.90)	7.28 (1.78)	0.99 (1.81)	3.25 (1.69)	6.90 (1.64)	2.25 (1.71)	0.81 (1.57)	0.87 (1.55)	0.35 (1.50)	0.28 (1.38)
Gifu	20	0.72 (2.01)	1.02 (1.82)	1.50 (1.89)	0.70 (1.88)	6.05 (1.92)	1.02 (2.04)	3.38 (1.91)	0.52 (1.96)	1.81 (1.77)	3.42 (1.75)	1.19 (1.81)	0.46 (1.70)	0.54 (1.73)	0.25 (1.47)	0.21 (1.36)
Hyogo	9	1.83 (2.04)	2.16 (2.22)	3.43 (2.28)	1.69 (1.95)	14.79 (1.91)	2.87 (1.85)	6.95 (2.12)	1.19 (1.61)	4.47 (1.84)	8.09 (1.75)	2.82 (1.72)	1.00 (1.68)	1.15 (1.64)	0.42 (1.60)	0.25 (1.39)
Total	89	1.24 (2.02)	1.69 (1.92)	2.51 (2.00)	1.13 (1.91)	10.33 (1.93)	1.73 (2.04)	5.87 (1.94)	0.83 (1.91)	2.76 (1.86)	5.47 (1.85)	1.87 (1.86)	0.65 (1.79)	0.75 (1.74)	0.32 (1.56)	0.25 (1.41)
(% of detected samples)		98.9	98.9	100	98.9	100	100	100	97.8	100	100	100	98.9	97.8	80.9	79.8

Supplementary table 6. Parameters used in the QSAR analysis for PBDEs and PCBs.

	No. of paired samples analyzed	Log P[a]	Log Kow	MW	Reference
**PCBs**					
TeCB #74	88	0.293	6.2	292.0	Hawker and Connell 1988
PeCB #99	88	0.306	6.4	326.4	Hawker and Connell 1988
PeCB #118	89	0.259	6.7	326.4	Hawker and Connell 1988
HxCB #146	88	0.303	6.9	360.9	Hawker and Connell 1988
HxCB #153	89	0.279	6.9	360.9	Hawker and Connell 1988
HxCB #164/#163	89	0.246	7.0	360.9	Hawker and Connell 1988
HxCB #138	89	0.281	6.4	360.9	Hawker and Connell 1988
HxCB #156	87	0.271	7.2	360.9	Hawker and Connell 1988
HpCB #182/#187	89	0.214	7.2	395.3	Hawker and Connell 1988
HpCB #180	89	0.190	7.4	395.3	Hawker and Connell 1988
HpCB #170	89	0.209	7.3	395.3	Hawker and Connell 1988
OCB #199	88	0.137	7.2	429.7	Hawker and Connell 1988
OCB #194	87	0.079	7.8	429.7	Hawker and Connell 1988
NCB #206	72	-0.137	8.1	464.2	Hawker and Connell 1988
DeCB #209	70	-0.297	8.2	498.6	Hawker and Connell 1988
**PBDEs**					
DiBDE #15	62	0.303	5.8	328.0	Palm et al. 2002
TrBDE #28	61	0.076	5.9	406.9	Braekevelt et al. 2003
TeBDE #47	89	0.070	6.8	485.8	Braekevelt et al. 2003
PeBDE #99	88	-0.012	7.3	564.7	Braekevelt et al. 2003
PeBDE #100	83	0.155	7.2	564.7	Braekevelt et al. 2003
HxBDE #153	89	0.066	7.9	643.6	Braekevelt et al. 2003
OBDE #197	88	-0.406	8.7	880.2	European Chemicals Bureau. 2003.
NBDE #207	84	-0.663	9.3	880.3	European Chemicals Bureau. 2003.
DeBDE #209	89	-0.751	9.9	959.1	European Chemicals Bureau. 2003.

[a]Log P is observed value. P is milk/serum partition coefficient.
